# Angiolymphoid hyperplasia with eosinophilia in a hand treated with a reverse digital island flap and artificial skin: a case report

**DOI:** 10.1186/s13256-019-2021-z

**Published:** 2019-03-27

**Authors:** Naohiro Oka, Shunji Nishimura, Hiroki Tanaka, Kazuhiko Hashimoto, Ryosuke Kakinoki, Masao Akagi

**Affiliations:** Department of Orthopedic Surgery, Kindai University Hospital, 377-2 Ohno-Higashi, Osaka-Sayama, Osaka 589-8511 Japan

**Keywords:** Angiolymphoid hyperplasia, Eosinophilia, Reverse palmar digital island flap, Collagen-based artificial skin

## Abstract

**Background:**

Angiolymphoid hyperplasia with eosinophilia is a rare nodular skin tumor characterized by eosinophilic invasion and vascular proliferation. Previous reports suggested that irritation and inflammation are the causative factors of this disease. Most cases of angiolymphoid hyperplasia with eosinophilia occur around the auricle, forehead, and scalp; the hand is rarely affected. Moreover, the tumor seldom presents as multiple nodules.

**Case presentation:**

A 67-year-old Japanese woman presented with a complaint of skin masses on her left thumb and index finger, which had gradually grown in size over the past few months. A biopsy was performed confirming a diagnosis of angiolymphoid hyperplasia with eosinophilia. The aponeurosis on her index finger was resected and tissue was reconstructed using a reverse palmar digital island flap harvested from the base of her index finger. The thumb lesion was also resected and covered with collagen-based artificial skin. Gradual progression of skin epithelialization followed by healing was noted 2 months after the surgery.

**Conclusion:**

Angiolymphoid hyperplasia with eosinophilia is a rare tumor; it is seldom seen in the hands. It is generally treated by surgical resection. It is important to resect a sufficiently large area of the tissue due to the possibility of relapse in some cases. Furthermore, appropriate reconstruction is mandatory after wide margin tumor resection.

## Introduction

Angiolymphoid hyperplasia with eosinophilia (ALHE) was first reported by Wells and Whimster in 1969 [1]. It is a rare skin tumor that occurs around the auricle, forehead, scalp, shoulder, chest, oral mucosa, and scrotum; the hand is rarely affected by this disease. ALHE is a benign tumor that presents with mild symptoms such as itching during the early stages. However, as the tumor grows, severe symptoms such as bleeding or pain may appear. Several studies indicated that the first choice of treatment of ALHE is surgical resection. However, the recurrence rate of this disease is reported to be as high as 41% [2], thus, necessitating the resection of a sufficiently large area in the affected region to ensure surgical success. Unfortunately, wide resection margins also result in large skin deformities, thereby reducing the daily living activities of the patient. Therefore, reconstruction is an additional important component of ALHE treatment.

Here we present a rare case of ALHE of the hand in a woman.

## Case presentation

A 67-year-old Japanese woman presented with complaints of a mass of skin on her left thumb and index finger that had been gradually increasing in size over the preceding few months. Her general condition was good (height, 147 cm; weight, 65 kg; heart rate, 62/minute; blood pressure, 136/72 mmHg, and body temperature, 36.3 °C). No abnormal breath or heart sounds were heard during auscultation. An abdominal examination revealed no tenderness, rigidity, or rebound, and her bowel sounds appeared normal. Neurological abnormalities such as absent or brisk deep tendon reflexes, muscle weakness, and hyperesthesia were not observed. In addition, no edema was present. According to the laboratory data, glycated hemoglobin (HbA1c) was high (8.9–9.4 over the previous 6 months); however, no abnormalities were noted in the other parameters. Furthermore, no abnormalities or signs of infection were observed in her urine analysis (Table 1).

**Table 1:**
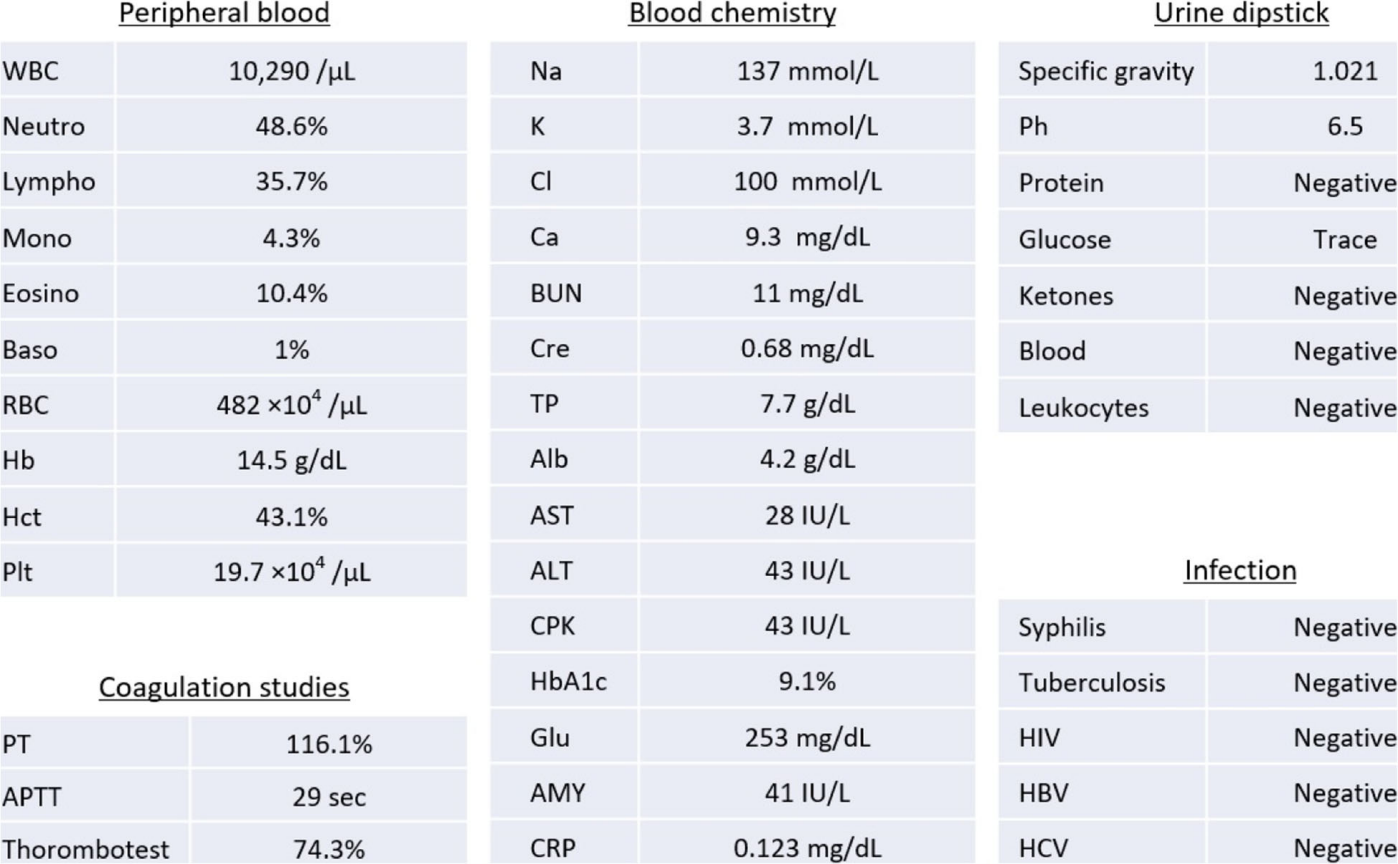
Laboratory findings at hospitalization

*Alb* albumin**,** *ALT* alanine aminotransferase, *AMY* amylase, *APTT* activated partial thromboplastin time, *AST* aspartate aminotransferase, *Baso* basophils, *BUN* blood urea nitrogen, *Ca* calcium, *Cl* chlorine, *CPK* creatine phosphokinase, *Cre* creatinine, *CRP* C-reactive protein, *Eosino* eosinophils, *Glu* glucose, *Hb* hemoglobin, *HbA1c* glycated hemoglobin, *HBV* hepatitis B virus, *Hct* hematocrit, *HCV* hepatitis C virus, *K* potassium, *Lympho* lymphocytes, *Mono* monocytes, *Na* sodium**,** *Neutro* neutrophils, *Plt* platelets, *PT* prothrombin time, *RBC* red blood cells, *TP* total protein, *WBC* white blood cells

She was under medication (hypoglycemic agents, antihypertensive drugs, and antihistamine drugs) for diabetes mellitus, hypertension, and allergic rhinitis, respectively. She had a history of smoking 10 cigarettes a day over the past 20 years. She did not drink alcohol, and there was no history of ALHE or other allergic diseases in her family. She was a housewife and was not involved in any specific occupation.

**Fig. 1:**
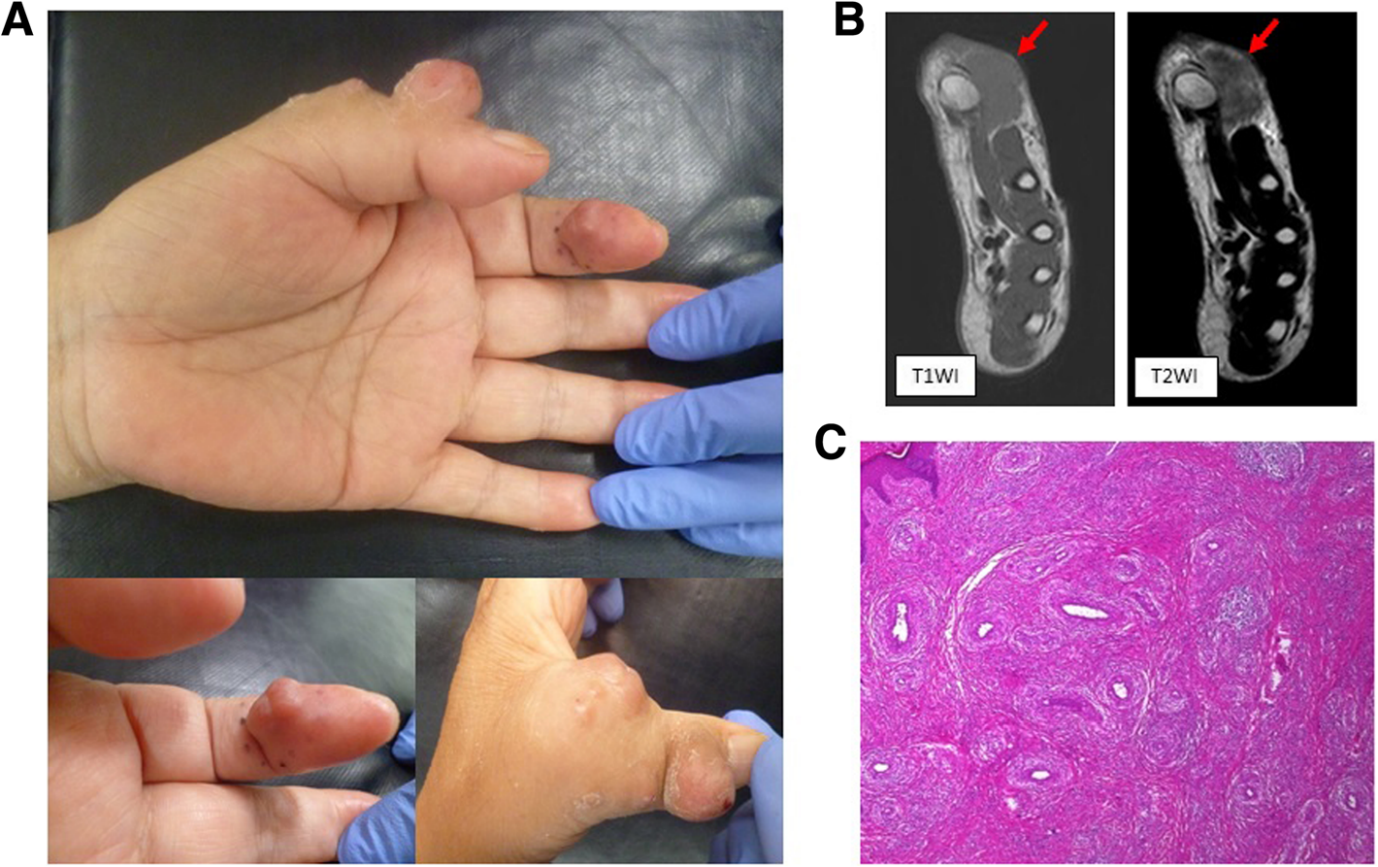
**a** Skin masses observed on the base of the left thumb and on the volar side of the index finger. **b** Magnetic resonance imaging of the lesion (red arrows) on the dorsal side of the thumb showed isointensity in T1-weighted images and high intensity in T2-weighted images. The tumor was confined to the skin. **c** Increase in the number of blood vessels in the dermal layer and eosinophilic infiltrates were noted around vascular endothelial cells. *T1WI* T1-weighted image, *T2WI* T2-weighted image

Several skin masses, each approximately 10 mm in diameter, were observed at the base and dorsum of her left thumb and on the volar side of her index finger (Fig. 1a). They were hard and reddish in color, with the majority of them presenting with a smooth, hairless surface and poor mobility.

**Fig. 2:**
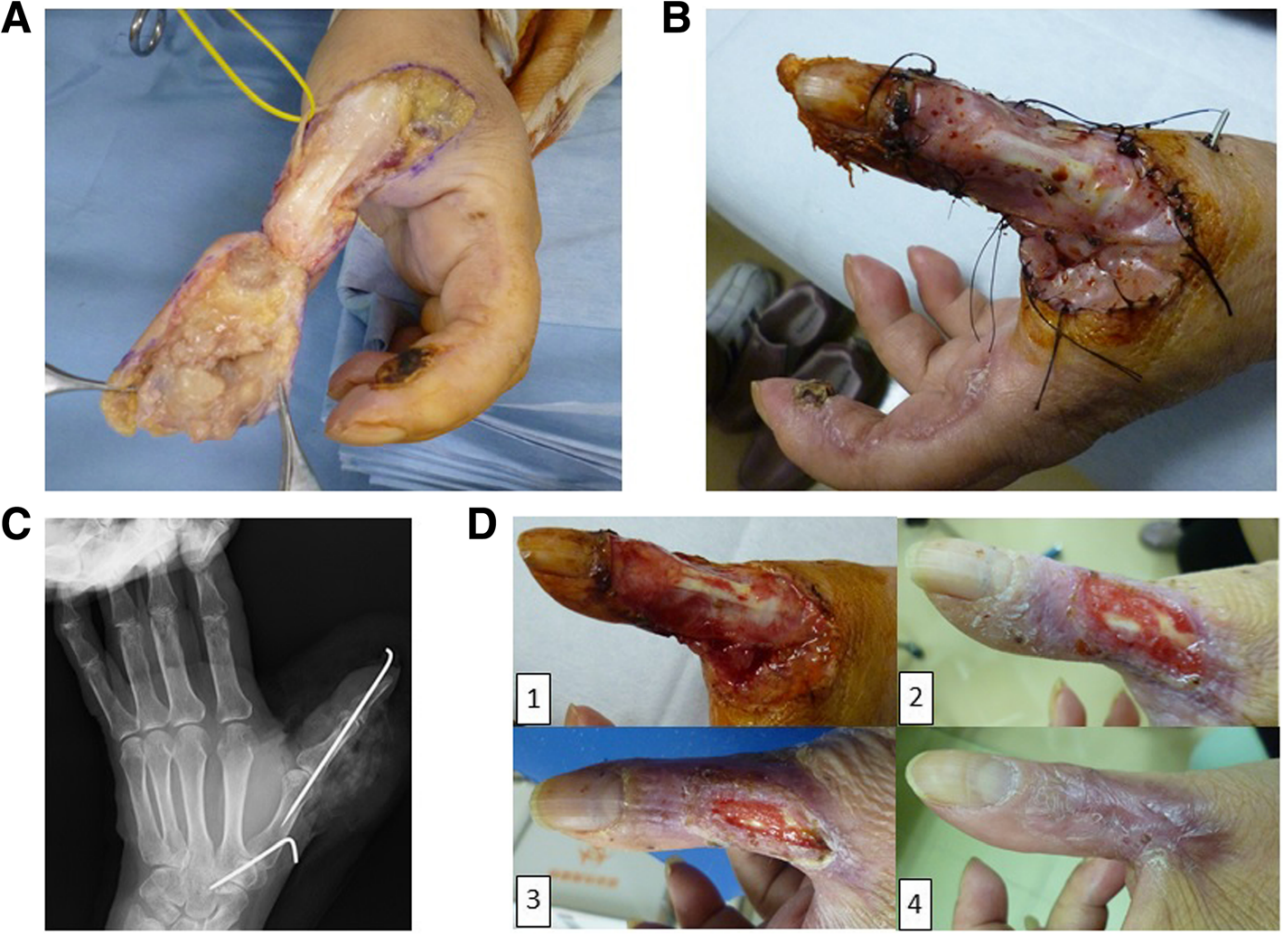
**a** The thumb lesion was excised along the margin of the tumor along with the aponeurosis. **b** After resection, the defect was covered with artificial skin using collagen. **c** Arthrodesis of the interphalangeal, metacarpophalangeal, and carpometacarpal joints of the thumb were performed using a Kirschner wire. **d** Gradually, epithelialization was noted after a few months

The masses were accompanied by pain, itching, and bleeding. A blood test indicated no inflammatory response; however, the eosinophil fraction was higher (11.8%) than the reference value. Magnetic resonance imaging revealed that the tumor was confined to the skin and had not extended to the thumb joint (Fig. 1b). A biopsy performed at the Department of Dermatology indicated signs of ALHE. She was then referred to the Department of Orthopedic Surgery for re-examination of the pathological condition, which was performed using tissue excised along the tumor margins on the distal phalanx of her index finger. The aponeurosis on the flexor digitorum superficialis, which was juxtaposed to the surface of the tumor, was also resected (Fig. 2a). Reconstruction was performed using a reverse palmar digital island flap harvested from the base of her index finger and the resected aponeurosis tissue was examined (Fig. 1c). Vascular proliferation in the dermis and infiltration of eosinophils around the surrounding vascular endothelial cells were noted. These findings were consistent with ALHE. Consequently, the lesions on her thumb and index finger were surgically excised using the tumor margins and aponeuroses as the resection range (Fig. 2a). As shown in Fig. 2b, the skin defect was covered with collagen-based artificial skin (Pelnac®, Gunze Co. Ltd., Ayabe, Japan). Subsequently, the interphalangeal and metacarpophalangeal joints of her thumb and the carpometacarpal joint were fixed using a Kirschner wire (Fig. 2c). She was prescribed loxoprofen sodium (180 mg/day) for approximately 10 days. The Kirschner wire was removed 2 weeks after the surgical procedure. A gradual progression of the epithelialization of the artificial skin was noted along with healing 2 months post-surgery (Fig. 2d). Mild contracture of the dorsal side of her thumb finger was observed after surgery; however, no hindrances in daily life activities were reported. Importantly, no tumor recurrence was noted at the 12-month follow-up.

## Discussion

ALHE is a nodular skin tumor associated with eosinophilia and vascular proliferation. Most cases occur on the auricle, forehead, and scalp, whereas rare cases, such as those involving the hand, account for only 4% of all ALHEs [2]. Table 2 describes the documented cases of ALHE involving hands. ALHE is difficult to cure [3]; the gold standard for treatment of ALHE is complete surgical resection. Due to the possibility of recurrence in some cases, complete resection of the tumor is necessary. Furthermore, functional reconstruction in conjunction with complete resection is essential for the treatment of ALHEs of the hand.

**Table 2:** Reported instances of angiolymphoid hyperplasia with eosinophilia in the hand

Study	Age	Sex	Lesion site	Number of lesions	Size	Symptoms	Treatment	Outcome	Systemic eosinophilia	Progression period
James M. Swinehart *et al.*, 1979 [20]	24	M	Lt.palm, subcutaneous	single	NA	NA	resection	local reccurence	+	8 months
Arnold M *et al.*, 1999 [21]	20	F	left arm and hand subcutaneous	multiple	5-10 mm	bleeding, pain	resection	no local reccurence, but another site	+	24 months
B. D. Krapohl *et al.*, 2003 [22]	33	F	Rt.palm and ring finger subcutaneous	multiple	NA	discoloration	resection	no recurrence in 3 months	NA	18 months
C Conill *et al.*, 2004 [23]	32	F	Rt.index and middle fingers subcutaneous and bone	multiple	NA	deformation of the nail, pain	radiation therapy	no recurrence in 9 years	–	NA
A. Satpathy, 2005 [24]	11	F	Rt.dorsum of hand subcutaneous	single	20 mm	itching	spontaneous resolution	no recurrence in 12 months	–	a month
H Ozcanli *et al.*, 2007 [25]	42	F	Rt.palm, middle and ring fingers subcutaneous	multiple	30-80 mm	itching, pain, disturbance of sensation	resection, laser treatment	no recurrence in 2 years	+	12 months
Nick Pappas *et al.*, 2010 [26]	18	F	Rt.palm subcutaneous	single	15-10 mm	no pain	resection	no recurrence in 1 year	–	a month
Mohammad M. Al-qattan *et al.*, 2017 [27]	32	F	Lt.palm subcutaneous	single	NA	NA	resection	no recurrence in 1 year	+	6 months

*F* female, *Lt.* left, *M* male, *NA* not available, *Rt.* right

On clinical examination, it is important to differentiate between ALHE and Kimura’s disease, using both laboratory and histological tests. Similar to AHLE, Kimura’s disease is classified as a type of eosinophilic dermatitis. Although eosinophilia was observed in the blood of the patient in the current report, systemic eosinophilia is generally rare. In addition, ALHE exhibits a slow growth rate and rarely presents with lymphadenopathy (5–20% of cases) [4]. Moreover, unlike in Kimura’s disease, the itching sensation is strong in AHLE. In many cases, immunoglobulin levels, such as that of immunoglobulin E, are normal. ALHE often involves neoangiogenesis and, accordingly, presents with plump, epithelioid endothelial cells and arteriovenous shunts [4]. Often, ALHE lesions are located in the dermis. Kimura’s disease is probably an allergic or autoimmune response, whereas ALHE is the result of a benign neoplasm of endothelial cells caused by inflammation or stimulation [5]. The cause of ALHE is unknown [6]. Some clinicians speculate that it develops in response to various infections, such as herpes virus 8 or human papillomavirus-6, or injury, such as excessive contact with the rim of eye glasses [7, 8]. Meanwhile, tumor growth is reported to occur in response to changes in hormone levels during pregnancy; however, there are no clear differences between men and women [9–12]. In the present case report, our patient had numerous skin injuries on her fingers other than that affected by the lesion. She reported that she frequently fed wild cats near her home and had received several scratches from the animals in the past, which may be related to the onset of ALHE in this case.

### Is it necessary to treat benign ALHE tumors?

ALHE does not develop complications during conservative therapy, and no cases of malignant transformation have been reported so far [5]. Yet, ALHE can be exacerbated by surgical stress; hence, refraining from excision without careful consideration is critical. Some cases of ALHE also recognize genetic mutation in T cell. According to Kempf *et al.*, ALHE is not a vascular lesion, but a type of CD4-positive T cell lymphoma [13–15]. Accordingly, careful observation is necessary, even in cases not involving surgical procedures. Early symptoms include itching and a throbbing sensation, but bleeding and pain are only observed as the tumor grows in size. Surgical resection may be indicated in the presence of more serious symptoms, such as pain, and if the patient wishes to undergo resection.

### Is there a cure for ALHE other than surgical resection?

In some cases the outcomes of administration of local and systemic corticosteroids to patients with AHLE were unclear. Furthermore, laser treatment has also been provided with unsatisfactory results [16, 17]. These treatments may be effective in elderly people and in situations where surgical procedures are contraindicated (such as in individuals using anticoagulants). The primary treatment choice for ALHE is surgical resection. However, the recurrence rate following surgical excision is high.

### What is critical for success in surgical resection?

Pathologically, ALHE involves vascular damage characterized by the presence of an unstable basement membrane and arteriovenous shunts [18, 19]. The removal of these abnormal vessels may be important during surgical resection. Furthermore, difficulties in identifying the margins of these lesions are believed to contribute to their high recurrence rate. Abnormal blood vessels related to ALHE often exist in the dermis; therefore, it is advisable to use natural anatomic barriers, such as the aponeurosis and fascia, as the resection margin. Several clinicians believe that ALHE resection should mirror the resection margin of malignant soft tissue tumors. It is important to examine the preoperative image before surgical treatment. In the present case study, both abnormal vessels and aponeurosis at the bottom of the tumor were excised. Recurrence is less likely when a wide resection with margins involving the dermis is performed. Reconstruction using a skin flap or artificial epithelium is recommended in cases where the deformity is large.

## Conclusion

ALHE is a rare, benign skin tumor characterized by the presence of vascular proliferation and eosinophil invasion, and is often treated conservatively. However, when accompanied by symptoms such as pain, surgical resection of the tumor is required. In such cases, removal of a sufficiently large area of resection is important to ensure a favorable outcome. In addition, adequate reconstruction is necessary to guarantee post-surgical functions.
